# Clinical Spectrum of Reversible Cerebral Vasoconstriction Syndrome With and Without Angiographic Vasoconstriction: A Retrospective Study of 84 Hospitalized Patients

**DOI:** 10.1002/brb3.71775

**Published:** 2026-09-17

**Authors:** Katsuya Takeuchi, Shoji Kikui, Yoshiki Matsumoto, Kenji Murakata, Hanako Sugiyama, Kuniko Ota, Daisuke Danno, Yoshihiro Kashiwaya, Takao Takeshima

**Affiliations:** ^1^ Department of Neurology and Headache Center Tominaga Hospital Osaka Japan

**Keywords:** clinical spectrum, magnetic resonance angiography, reversible cerebral vasoconstriction syndrome, thunderclap headache, vasoconstriction

## Abstract

**Objective:**

To determine whether patients with reversible cerebral vasoconstriction syndrome (RCVS) without demonstrable vasoconstriction differ clinically from those with angiographically confirmed vasoconstriction.

**Methods:**

We retrospectively studied consecutive patients with RCVS hospitalized at a tertiary headache center in Japan between February 2011 and March 2026. Patients were classified according to the presence or absence of angiographically demonstrable vasoconstriction on magnetic resonance angiography. Demographic characteristics, clinical features, surrogate markers of disease severity, treatment patterns, triggering factors, neuroimaging abnormalities, and outcomes were compared between groups.

**Results:**

Eighty‐four patients were included: 61 with demonstrable vasoconstriction and 23 without. The two groups did not differ significantly in age (median, 51 vs. 53 years; *p* = 0.213), female predominance (80% vs. 78%; *p* = 1.000), or migraine history (59% vs. 57%; *p* = 1.000). Clinical features were similar, including the number of thunderclap headache episodes (median, 4 vs. 4; *p* = 0.577) and the interval from the first to the last thunderclap headache (median, 6 vs. 6 days; *p* = 0.460). Time to hospitalization, length of hospital stay, calcium channel blocker use (98% vs. 100%; *p* = 1.000), neuroimaging abnormalities (8% vs. 9%; *p* = 1.000), and persistent neurological sequelae (2% vs. 0%; *p* = 1.000) were also comparable.

**Conclusion:**

Patients without demonstrable vasoconstriction exhibited clinical characteristics, severity indicators, management, and outcomes similar to those with confirmed vasoconstriction. These findings suggest that the two groups may share a common clinical spectrum; however, confirmation in larger prospective studies is warranted.

## Graphical abstract

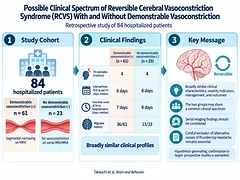

## 1 Introduction

Reversible cerebral vasoconstriction syndrome (RCVS) is a clinical syndrome characterized by recurrent thunderclap headaches and multifocal reversible constriction of the cerebral arteries (Calabrese et al. 2007). RCVS predominantly affects middle‐aged women, and is frequently associated with a history of migraine, exposure to vasoactive substances, and various physiological and emotional triggers (Ducros et al. 2007; Ducros 2012). Although the clinical course is generally self‐limiting with a favorable prognosis, complications, such as cerebral infarction, intracranial hemorrhage, and posterior reversible encephalopathy syndrome, may occur (Ducros et al. 2007).

The diagnosis of RCVS traditionally relies on the demonstration of segmental cerebral vasoconstriction and its reversibility on vascular imaging (Calabrese et al. 2007; Ducros 2012). However, vasoconstriction is a dynamic and time‐dependent process that may not be detectable based on imaging timing (Marder et al. 2012). Therefore, in clinical practice, patients who present with typical features of RCVS but lack demonstrable vasoconstriction on magnetic resonance angiography (MRA) are common.

However, the clinical significance of these findings remains unclear. Whether patients without demonstrable vasoconstriction represent a distinct entity or fall within the same clinical spectrum as those with vasoconstriction remains to be elucidated. This issue is of particular importance in headache management because failure to recognize RCVS can result in underdiagnosis or delayed treatment.

Furthermore, previous studies were mainly conducted in Western populations, and data from Asian cohorts, particularly from tertiary headache centers, are limited. Given the potential differences in the clinical presentation and disease severity across populations, further investigation is warranted.

In this study, we aimed to compare the clinical characteristics, disease severity, management, and outcomes of patients with and without angiographically confirmed vasoconstriction in a cohort of hospitalized patients with RCVS at a tertiary headache center in Japan. We hypothesized that patients without demonstrable vasoconstriction would exhibit clinical features similar to those with confirmed vasoconstriction, raising the possibility that the two groups may lie along a common clinical spectrum.

## 2 Methods

### 2.1 Study Design and Participants

This retrospective observational study included consecutive patients with RCVS who were hospitalized at a tertiary headache center in Japan (Tominaga Hospital) between February 2011 and March 2026. A total of 84 patients were included: 61 patients with confirmed RCVS and 23 patients who fulfilled the clinical criteria for probable RCVS without demonstrable vasoconstriction on MRA.

### 2.2 Diagnostic Criteria

The diagnosis of RCVS was based on the criteria proposed by Ducros (2012). Acute headache attributed to RCVS and probable RCVS were classified according to the International Classification of Headache Disorders, third edition (Headache Classification Committee of the International Headache Society [IHS] 2018). All diagnoses were independently confirmed by at least two headache specialists certified by the Japanese Headache Society.

RCVS was classified as secondary when a recognized associated condition or exposure temporally related to RCVS onset was identified, including vasoactive or potentially causative medications, the postpartum state, Cushing's syndrome, or recent neurosurgical or endovascular procedures. Cases without identifiable associated conditions or exposures were classified as idiopathic RCVS.

### 2.3 Neuroimaging Assessment

In routine clinical practice, the treating headache specialist suspected RCVS based on the clinical history and neurological examination and obtained MRI/MRA for diagnostic evaluation. The presence or absence of vasoconstriction on MRA was independently assessed by a certified headache specialist who was not involved in the clinical care of the patients and a board‐certified neuroradiologist, both of whom were blinded to the clinical information. The inter‐rater agreement between the two blinded reviewers was assessed using Cohen's *κ* coefficient. For the final imaging classification, the imaging findings and clinical information were reviewed at a multidisciplinary neurology conference involving three certified headache specialists and one board‐certified neuroradiologist, and the presence or absence of vasoconstriction was determined by consensus. In patients in whom vasoconstriction was identified, its reversibility was verified using follow‐up MRA within 3 months. Given that vasoconstriction is a dynamic and time‐dependent phenomenon, patients without demonstrable vasoconstriction on initial imaging may have vasoconstriction that was not captured at the time of examination (Ducros 2012; Marder et al. 2012). The timing of the initial MRI/MRA was defined as the interval from the onset of the first thunderclap headache to the first MRI/MRA. All patients underwent standardized serial MRI/MRA regardless of the presence or absence of vasoconstriction, including examinations at admission, during hospitalization, and at least once after discharge at ≥ 1 month after symptom onset, ensuring a minimum of three imaging assessments per patient. Patients without vasoconstriction on follow‐up imaging and without recurrent thunderclap headache were discharged from further outpatient follow‐up. Patients with persistent neurological sequelae or neuroimaging abnormalities, such as cerebral infarction, continued to receive clinical follow‐up as required.

### 2.4 Treatment

All patients were hospitalized and received standard treatment for RCVS regardless of the presence of vasoconstriction. In accordance with the Japanese clinical practice guidelines, calcium channel blockers, primarily lomerizine (10–20 mg/day), or verapamil (typically 240 mg/day), were administered at the discretion of the treating physicians (Araki et al. 2025).

### 2.5 Data Collection

Clinical data were extracted from the electronic medical records, including age at onset, sex, time from onset of thunderclap headache to initial MRI/MRA, time from onset of thunderclap headache to hospitalization, length of hospital stay, number of thunderclap headache episodes, interval from the first to the last thunderclap headache, history of migraine, triggering factors, presence of secondary RCVS, medical history, presence of a hypertensive surge at onset, and treatment details.

### 2.6 Statistical Analysis

The clinical characteristics of patients with confirmed RCVS were compared to those of patients without demonstrable vasoconstriction. Continuous variables are expressed as medians (interquartile range) and compared using the Mann–Whitney *U* test. Categorical variables are expressed as numbers (%) and compared using Fisher's exact test, as appropriate. For variables with more than two categories (e.g., triggering factors), Fisher's exact test with the Freeman–Halton extension was used.

Effect estimates with 95% confidence intervals (CIs) were calculated to quantify the magnitude and precision of the between‐group differences. For continuous variables, between‐group differences were estimated using the Hodges–Lehmann estimator, with 95% CIs obtained using percentile bootstrap resampling (10,000 resamples). For categorical variables, absolute risk differences with Newcombe–Wilson 95% CIs were calculated. To account for multiple comparisons, *p‐*values were adjusted using the Benjamini–Hochberg false discovery rate (FDR) procedure.

All tests were two‐tailed, and statistical significance was set at *p* < 0.05. Statistical analyses were performed using IBM SPSS Statistics for Windows (version 24.0; IBM Corp., Armonk, NY).

### 2.7 Ethical Considerations

This study was approved by the Ethics Committee of Tominaga Hospital (approval no. 120184) and conducted in accordance with the Declaration of Helsinki. Owing to the retrospective nature of the study, the requirement for written informed consent was waived in accordance with institutional policy.

## 3 Results

### 3.1 Patient Characteristics

A total of 84 patients were included: 61 with confirmed RCVS and 23 without demonstrable vasoconstriction. Both groups comprised predominantly middle‐aged females. No significant differences were observed between the groups in terms of age (median, 51 vs. 53 years; *p* = 0.213) or sex distribution (80% vs. 78%; *p* = 1.000).

A history of migraine was present in 59% of patients with vasoconstriction and 57% of those without, with no significant difference (*p* = 1.000). The proportion of idiopathic cases was comparable between the groups (80% vs. 87%, *p* = 0.750). Secondary RCVS was observed in 12 (20%) patients with vasoconstriction and 3 (13%) without. Drug‐induced cases were the most frequent, whereas other causes, including Cushing's syndrome and postpartum onset, were rare. Similarly, the frequency of hypertensive surges at onset did not differ significantly between the groups (7% vs. 13%, *p* = 0.386). The detailed baseline characteristics are summarized in Table 1.

**TABLE 1:** Baseline characteristics and triggering factors in patients with reversible cerebral vasoconstriction syndrome with and without demonstrable vasoconstriction.

	Vasoconstriction (+) (*n* = 61)	Vasoconstriction (–) (*n* = 23)	Effect estimate (95% CI)	*p*‐value	FDR‐adjusted *p*
Age, years, median (IQR)	51 (38−57)	53 (49−57)	−3 years (−8 to 1)	0.213	1.000
Female sex, *n* (%)	49 (80)	18 (78)	+2.1 pp (−14.7 to +23.8)	1.000	1.000
History of migraine, *n* (%)	36 (59)	13 (57)	+2.5 pp (−19.3 to +25.3)	1.000	1.000
Idiopathic RCVS, *n* (%)	49 (80)	20 (87)	−6.6 pp (−21.0 to +14.1)	0.750	1.000
Secondary RCVS, *n* (%)	12^a^ (20)	3^b^ (13)	+6.6 pp (−14.1 to +21.0)	0.750	1.000
Hypertensive surge at onset, *n* (%)	4 (7)	3 (13)	−6.5 pp (−26.0 to 6.0)	0.386	1.000
Triggering factors, *n* (%)					
Valsalva‐like maneuver	18 (30)	7 (30)	−0.9 pp (−23.7 to +18.4)		
Bathing and/or showering	9 (15)	5 (22)	−7.0 pp (−28.3 to +9.3)		
Physical exertion	6 (10)	4 (17)	−7.6 pp (−28.0 to +6.9)		
Sexual activity	6 (10)	1 (4)	+5.5 pp (−12.0 to +16.1)		
Emotional stress	5 (8)	1 (4)	+3.8 pp (−13.4 to +14.1)		
No identifiable trigger	17 (28)	5 (22)	+6.1 pp (−16.2 to +23.4)		
Overall comparison				0.822	1.000

*Note*: Data are presented as median (interquartile range) or number (%). For continuous variables, between‐group differences were estimated using the Hodges–Lehmann estimator, with 95% confidence intervals obtained using percentile bootstrap resampling (10,000 resamples). For categorical variables, absolute risk differences with Newcombe–Wilson 95% confidence intervals (CIs) were calculated. *p*‐values were calculated using the Mann–Whitney *U* test for continuous variables and Fisher's exact test for categorical variables. For triggering factors, the overall *p*‐value was calculated using Fisher's exact test with the Freeman–Halton extension. The *p*‐values were adjusted for multiple comparisons using the Benjamini–Hochberg false discovery rate procedure.

Abbreviations: IQR, interquartile range; MTX, methotrexate; RCVS, reversible cerebral vasoconstriction syndrome; SNRI, serotonin‐norepinephrine reuptake inhibitors; SSRI, selective serotonin reuptake inhibitor.

^a^ Drug induced 7 (SSRI 3, Triptan 2, SNRI 1, Etanercept 1), Postpartum 2, Cushing syndrome 1, recent neurosurgical or endovascular procedures 2.

^b^ Drug induced 3 (Triptan 2, Methotrexate 1).

### 3.2 Serial Neuroimaging Findings

Among the 61 patients ultimately classified as having demonstrable vasoconstriction, vasoconstriction was identified on the initial MRI/MRA in 39 patients and was first detected on the second MRI/MRA in 22 patients. None of the 23 patients classified as having no demonstrable vasoconstriction showed vasoconstriction on any of the serial MRI/MRA examinations, including follow‐up imaging performed post‐discharge. At the patient level, the inter‐rater agreement between the two blinded reviewers was 88.1% (74/84), with a Cohen's *κ* coefficient of 0.65. The *κ* coefficients were 0.64 for the initial MRI/MRA and 0.84 for the second MRI/MRA scans. Complete agreement was observed in the third MRI/MRA.

### 3.3 Clinical Features and Severity Indicators

No significant differences were observed in key clinical features between the groups. The number of thunderclap headache episodes was similar (median 4 vs. 4, *p* = 0.577), as was the interval from the first to the last thunderclap headache (median 6 vs. 6 days, *p* = 0.460).

The time from the onset of thunderclap headache to hospitalization (median, 7 vs. 6 days; *p* = 0.244) was similar between the groups. The median time from the onset of thunderclap headache to the initial MRI/MRA was 4 days (IQR: 3–8) in the vasoconstriction‐positive group and 2 days (IQR: 0.5–6.5) in the vasoconstriction‐negative group, with no statistically significant difference (*p* = 0.091). The length of hospital stay was also comparable (median, 12 vs. 11 days; *p* = 0.852) (Table 2).

**TABLE 2:** Clinical features, disease severity, management, and outcomes in patients with reversible cerebral vasoconstriction syndrome with and without demonstrable vasoconstriction.

	Vasoconstriction (+) (*n* = 61)	Vasoconstriction (−) (*n* = 23)	Effect estimate (95% CI)	*p*‐value	FDR‐adjusted *p*
Clinical features					
Number of TH episodes, median (IQR)	4 (3–6)	4 (2–7.5)	0 (−2 to 2)	0.577	1.000
Interval from the first to the last TH, days, median (IQR)	6 (4–11)	6 (2–12)	1 day (−2 to 3)	0.460	1.000
Severity indicators					
Time from onset of TH to initial MRI/MRA, days, median (IQR)	4 (3–8)	2 (0.5–6.5)	2 days (0–3)	0.091	1.000
Time from onset of TH to hospitalization, days, median (IQR)	7 (4–11)	6 (3–10)	1 day (−1 to 4)	0.244	1.000
Length of hospital stay, days, median (IQR)	12 (8–14)	11 (9.5–15.5)	0 day (−3 to 3)	0.852	1.000
Treatment					
Calcium channel blocker use, *n* (%)	60 (98)	23 (100)	−1.6 pp (−8.7 to +12.7)	1.000	1.000
Outcomes					
Neuroimaging abnormalities, *n* (%)	5^a^(8)	2^b^ (9)	−0.5 pp (−19.2 to +11.0)	1.000	1.000
Persistent neurological sequelae, *n* (%)	1^c^(2)	0 (0)	+1.6 pp (−12.7 to +8.7)	1.000	1.000
Recurrence of TH	1 (2)	0 (0)	+1.6 pp (−12.7 to +8.7)	1.000	1.000

*Note*: Data are presented as median (interquartile range) or number (%). For continuous variables, between‐group differences were estimated using the Hodges–Lehmann estimator, with 95% confidence intervals obtained using percentile bootstrap resampling (10,000 resamples). For categorical variables, absolute risk differences with Newcombe–Wilson 95% confidence intervals (CIs) were calculated. *p*‐values were calculated using the Mann–Whitney *U* test for continuous variables and Fisher's exact test for categorical variables. The *p*‐values were adjusted for multiple comparisons using the Benjamini–Hochberg false discovery rate procedure.

Abbreviations: CI, confidence interval; IQR, interquartile range; MRA, magnetic resonance angiography; MRI, magnetic resonance imaging; pp, percentage points; TH, thunderclap headache.

^a^ Cerebral infarction 3, Cortical subarachnoid hemorrhage 2.

^b^ Cerebral infarction 1, Cortical subarachnoid hemorrhage 1.

^c^ Mild hemiparesis.

### 3.4 Treatment

Calcium channel blockers were used by nearly all patients in both groups, with no significant differences in the usage rates (98% vs. 100%, *p* = 1.000) (Table 2).

### 3.5 Triggering Factors

Valsalva‐like maneuvers and the absence of identifiable triggers were common to both groups of patients. The overall distribution of triggering factors did not differ significantly between the groups (*p* = 0.822) (Table 1).

### 3.6 Neuroimaging Abnormalities and Outcomes

Neuroimaging abnormalities were observed in 8% of patients with vasoconstriction and 9% of those without, with no significant difference (*p* = 1.000). Persistent neurological sequelae were rare, occurring in only one patient (2%) in the vasoconstriction group and none of the patients without vasoconstriction (*p* = 1.000). Although persistent neurological sequelae are rare, one patient in the vasoconstriction group developed a neurological deficit, which has been reported separately in a detailed case report (Kikui et al. 2026). The recurrence of thunderclap headache was infrequent in both groups, with no significant differences (Table 2).

### 3.7 Summary of Comparisons

Overall, no significant differences were observed between the patients with and without vasoconstriction in terms of demographic characteristics, clinical features, severity indicators, management, triggering factors, or outcomes. None of the between‐group comparisons were statistically significant after FDR adjustment.

## 4 Discussion

In this study, we compared patients with and without angiographically demonstrable vasoconstriction in a cohort of hospitalized patients with RCVS at a tertiary headache center in Japan. No significant differences were found between the two groups in terms of demographic characteristics, clinical features, surrogate markers of disease severity, management, triggering factors, neuroimaging abnormalities, or outcomes. These findings suggest that the absence of demonstrable vasoconstriction may not necessarily indicate a clinically distinct or milder subgroup.

**FIGURE 1:**
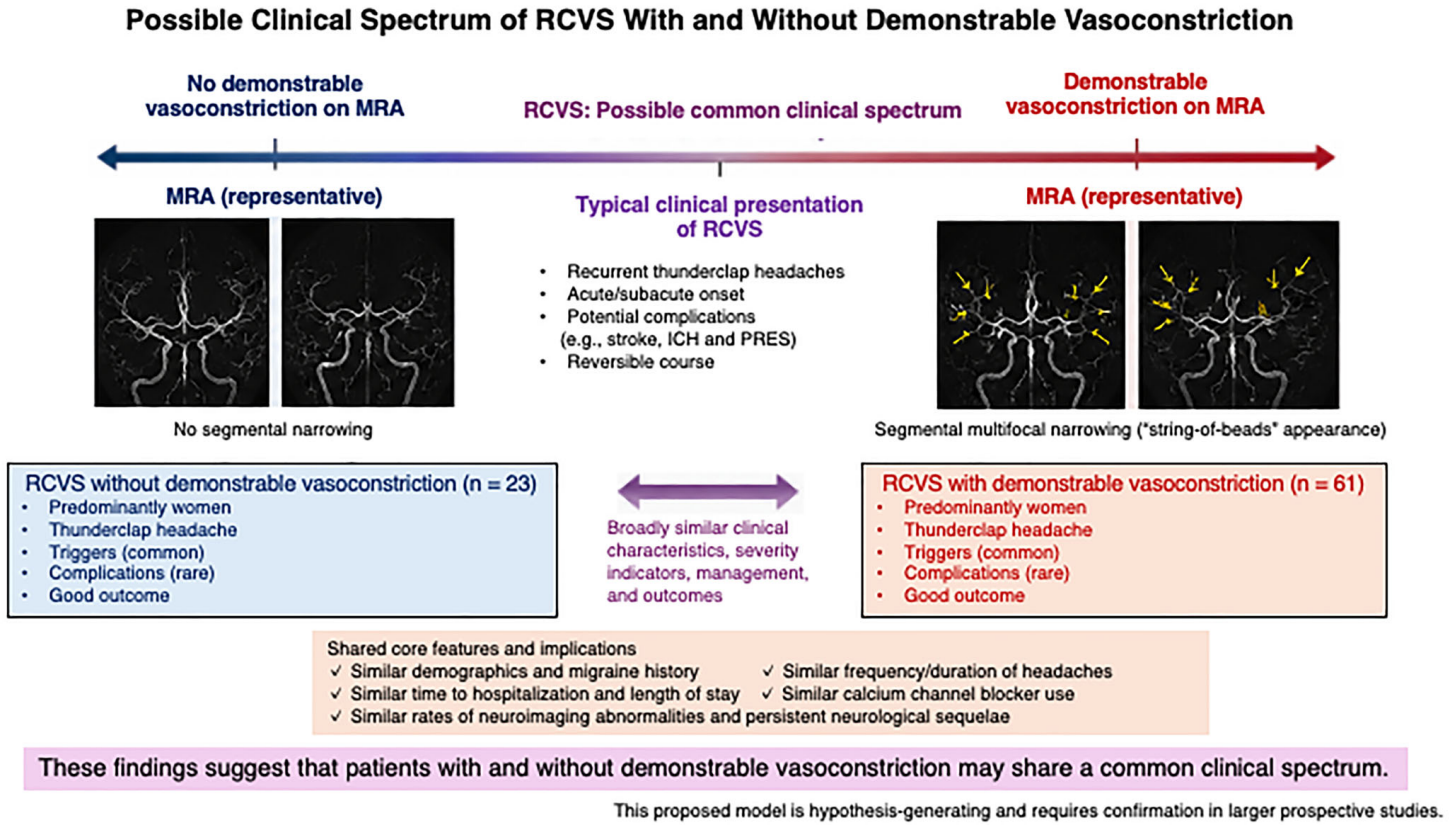
Possible clinical spectrum of reversible cerebral vasoconstriction syndrome with and without demonstrable vasoconstriction on magnetic resonance angiography. Schematic illustration of the proposed spectrum model of reversible cerebral vasoconstriction syndrome (RCVS). Patients without demonstrable vasoconstriction showed clinical characteristics, severity indicators, management, and outcomes similar to those with angiographically confirmed vasoconstriction. These findings suggest that the two groups may share a common clinical spectrum of symptoms. However, this interpretation is hypothesis‐generating and requires confirmation in larger, prospective studies. ICH, intracranial hemorrhage; MRA, magnetic resonance angiography; PRES, posterior reversible encephalopathy syndrome; RCVS, reversible cerebral vasoconstriction syndrome.

A schematic representation of this concept is presented in Figure 1. Taken together, our findings are compatible with the possibility that patients with and without visible vasoconstriction may lie along a common clinical spectrum rather than representing entirely separate categories of patients. This interpretation should be regarded as hypothesis‐generating and requires confirmation in larger, prospective studies. These observations also suggest that angiographic findings should be interpreted in the context of the overall clinical presentation rather than used in isolation.

RCVS is traditionally defined as reversible segmental vasoconstriction of the cerebral arteries; however, vasoconstriction is a dynamic and time‐dependent phenomenon, and its detectability depends on the timing of the vascular imaging (Ducros 2012; Marder et al. 2012). Because vasoconstriction may not be apparent early in the disease course and may become detectable only on subsequent imaging, the timing of vascular imaging and the clinical diagnostic context may influence the recognition and classification of RCVS, particularly in patients without initially demonstrable vasoconstriction (Ducros 2012; Marder et al. 2012). Several mechanisms may explain the absence of detectable vasoconstriction, despite the typical clinical features. First, vasoconstriction might not have been captured at the time of imaging because of its transient nature (Ducros 2012; Marder et al. 2012). Second, vasoconstriction may predominantly involve distal or small‐caliber vessels below the spatial resolution of conventional MRA. Third, transient dysregulation of the cerebral vascular tone, mediated by sympathetic overactivity and vasoactive substances, may produce a characteristic clinical phenotype, even in the absence of overt large vessel narrowing (Singhal et al. 2002; Singhal 2004). These mechanisms are not mutually exclusive.

Our findings have potential clinical implications. Patients presenting with recurrent thunderclap headaches and typical triggers may pose a diagnostic challenge when angiographic vasoconstriction is not observed. Our results suggest that the absence of demonstrable vasoconstriction, particularly on initial imaging, should be interpreted in conjunction with the overall clinical presentation and serial imaging findings. However, the careful exclusion of alternative causes of thunderclap headache remains essential to avoid the overdiagnosis of RCVS.

The overall clinical profile of our cohort, including the predominance of middle‐aged women, frequent history of migraine, and generally favorable outcomes, is consistent with previous reports (Ducros et al. 2007; Ducros 2012). The comparable distribution of triggering factors and similar treatment patterns may further suggest shared clinical features between these two groups.

A strength of this study is the use of serial MRI/MRA assessments in all patients, which may reduce, although not eliminate, the risk of misclassification related to the dynamic nature of vasoconstriction. Notably, patients without demonstrable vasoconstriction remained negative on at least three serial MRI/MRA examinations, including follow‐up imaging performed after their discharge.

Importantly, the absence of statistically significant differences should not be interpreted as evidence of equivalence between the two groups. Given the modest sample size, particularly in the vasoconstriction‐negative group, our findings should be considered as exploratory and hypothesis‐generating. The relatively wide CIs for several effect estimates also indicate substantial uncertainty, and clinically meaningful between‐group differences cannot be excluded. Nevertheless, the consistency of the findings across multiple clinical domains suggests that the two groups may share similar clinical profiles.

This study had certain limitations. First, although two independent reviewers assessed the MRA findings while blinded to the clinical information and showed substantial inter‐rater agreement, the final imaging classification was determined by consensus with access to clinical information. Therefore, the possibility of confirmation bias in the final adjudication cannot be excluded. Second, transient vasoconstriction or involvement of distal or small‐caliber vessels below the spatial resolution of conventional MRA may have remained undetected despite serial imaging; therefore, misclassification cannot be completely excluded. Third, although no significant differences were observed, the sample size was relatively modest, and the study may have been underpowered to detect small but clinically meaningful differences in the outcomes. Finally, as this was a retrospective single‐center study conducted at a tertiary referral center, a selection bias cannot be excluded. Furthermore, because patients without demonstrable vasoconstriction were classified according to the clinical criteria for probable RCVS, the possibility that this group included RCVS mimics, such as primary thunderclap headache, cannot be completely excluded despite serial MRI/MRA assessments and specialist review. Such diagnostic misclassification could have attenuated the true between‐group differences, particularly because patients in the vasoconstriction‐negative group were selected based on clinical features resembling RCVS. Therefore, the observed similarities between the groups should be interpreted cautiously.

## 5 Conclusion

In this single‐center retrospective cohort study, patients with and without demonstrable vasoconstriction showed broadly similar clinical characteristics, severity indicators, management, and outcomes. These findings suggest that patients without demonstrable vasoconstriction may share clinical features with those with confirmed vasoconstriction and may lie on a common clinical spectrum. However, given the modest sample size and inherent diagnostic uncertainty, these findings should be considered exploratory and require confirmation in larger, prospective studies. In patients with a typical clinical presentation, the absence of demonstrable vasoconstriction on MRA should not preclude the consideration of RCVS. Serial imaging findings and careful exclusion of alternative causes of thunderclap headache should be considered when making a diagnosis.

## Author Contributions

**Katsuya Takeuchi**: conceptualization, data curation, investigation, methodology, Writing – original draft, Writing – review and editing. **Kenji Murakata**: investigation, data curation, validation, writing – review and editing. **Daisuke Danno**: investigation, methodology, validation, writing – review and editing. **Yoshihiro Kashiwaya**: investigation, resources, validation, writing – review and editing. **Takao Takeshima**: supervision, methodology, validation, writing – review and editing. **Shoji Kikui**: conceptualization, data curation, formal analysis, methodology, project administration, supervision, writing – original draft, writing – review and editing. **Yoshiki Matsumoto**: investigation, data curation, validation, writing – review and editing. **Hanako Sugiyama**: investigation, data curation, validation, writing – review and editing. Kuniko Ota: investigation, data curation, writing – review and editing.

## Funding

The authors have nothing to report.

## Conflicts of Interest

Shoji Kikui has received speaking fees from Amgen, Daiichi Sankyo, Eli Lilly, and Otsuka Pharmaceutical. Daisuke Danno has received speaking fees from Amgen, Daiichi Sankyo, Eli Lilly, and Otsuka Pharmaceutical. Takao Takeshima has received speaking fees from Amgen, Daiichi Sankyo, Eisai, Eli Lilly, and Otsuka Pharmaceutical. He also serves on the editorial boards of the Japanese Headache Society and the Japanese Society for Neurology. These relationships had no impact on the results or conclusions of the present study. The other authors declare no conflicts of interest.

## Data Availability

The data underlying this study are not publicly available due to institutional and ethical restrictions but may be made available from the corresponding author upon reasonable request.
